# Transcriptional Regulation of Emergency Granulopoiesis in Leukemia

**DOI:** 10.3389/fimmu.2018.00481

**Published:** 2018-03-12

**Authors:** Shirin Hasan, Afsar R. Naqvi, Asim Rizvi

**Affiliations:** ^1^Feinberg School of Medicine, Northwestern University, Chicago, IL, United States; ^2^Department of Periodontics, University of Illinois at Chicago, Chicago, IL, United States; ^3^Department of Biochemistry, Aligarh Muslim University, Aligarh, India

**Keywords:** emergency granulopoiesis, Fanconi DNA repair pathway, infections, leukemia, neutropenia, neutrophils

## Abstract

Neutropenic conditions are prevalent in leukemia patients and are often associated with increased susceptibility to infections. In fact, emergency granulopoiesis (EG), a process regulating neutrophil homeostasis in inflammatory conditions and infections, may occur improperly in leukemic conditions, leading to reduced neutrophil counts. Unfortunately, the mechanisms central to dysfunctional EG remain understudied in both leukemia patients and leukemic mouse models. However, despite no direct studies on EG response in leukemia are reported, recently certain transcription factors (TFs) have been found to function at the crossroads of leukemia and EG. In this review, we present an update on TFs that can potentially govern the fate of EG in leukemia. Transcriptional control of Fanconi DNA repair pathway genes is also highlighted, as well as the newly discovered role of Fanconi proteins in innate immune response and EG. Identifying the TFs regulating EG in leukemia and dissecting their underlying mechanisms may facilitate the discovery of therapeutic drugs for the treatment of neutropenia.

## Introduction

Bacterial and fungal infections are a leading cause of morbidity and mortality in acute leukemia patients (1, 2). A recent study reported that 71.9% of chronic lymphocytic leukemia (CLL) patients developed infections with a mortality rate of 37.5% (3). Abnormal proliferation of myeloid cells occurs and immature leukocytes accumulate in bone marrow in a cancer microenvironment that can also inhibit antigen-specific T cell response (4). Therefore, leukemia patients are particularly at a high risk for infectious complications. Highly intensive chemotherapy results in prolonged neutropenia, rendering the patients extremely susceptible to microbial infections (5). Prolonged periods of neutropenia proportionately increase the risk of severe infections, which can be exacerbated with the relapse of disease (6, 7).

Neutrophils are key mediators of the early inflammatory response at the time of an infection, and reduced neutrophil counts can lead to life-threatening infections (8). Neutrophil homeostasis is differentially regulated during steady state and infectious episodes. During infections or inflammation, circulating neutrophils become significantly elevated in a process called emergency granulopoiesis (EG). This process involves enhanced generation of neutrophils in the bone marrow through increased myeloid progenitor cell proliferation (9). Neutrophil mobilization is also increased in response to elevation in circulatory granulocyte colony stimulating factor (G-CSF) levels resulting in effective clearance of bacterial and fungal pathogens (10).

Execution of EG occurs in four different stages: (i) it commences with an increase in the peripheral neutrophil count due to vascular demargination and releases from the bone marrow mediated by disruption of CXCL12/CXCR4 signaling (11–15). This phase is accompanied by (ii) *de novo* generation of neutrophils from increased myeloid progenitor cell proliferation (9) and (iii) their accelerated differentiation by S-phase shortening of the cell cycle stabilized by the Fanconi pathway (16) followed by (iv) termination of EG response, which is partly mediated by interferon regulatory factor (IRF8) (17). A recent study demonstrated that, in cancer chemotherapy-induced neutropenia (CCIN), the neutrophils generated during EG response were functionally immature in both humans and mouse models and displayed weak bactericidal activity (18). Studies evaluating the defects in completion of the EG response during leukemic conditions are scarce; however, certain transcription factors (TFs) have been identified, which share an overlapping role in leukemia and innate immunity. The purpose of this review is to characterize the role of these TFs in leukemia and their link to the EG response. Recently discovered role of the Fanconi DNA repair pathway in innate immunity will also be discussed.

## Regulation of Emergency Granulopoiesis

Eemergency granulopoiesis is regulated by various endogenous and exogenous factors. Our knowledge of endogenous factors, predominantly transcriptional regulators, has increased significantly over the past decade. Various TFs play a pivotal role in modulating both EG and leukemia development. Recent studies have shown that dysregulation of these TFs leads to perturbed granulopoiesis along with an aggravation of leukemic state. The role of TFs in normal hematopoiesis, EG, and leukemogenesis is discussed in the following section. Table 1 presents a list of the TFs discussed here.

**Table 1:** Transcription factors with intersecting roles in EG and leukemia.

Transcription Factors	Functions
HOXA10	Postnatal hematopoietic development and HSC self-renewal (19) Development of lymphoid/erythroid/megakaryocyte cells (21) AML development (26) Termination of EG response by activating TRIAD1 (32)

CEBP-β	Granulocyte proliferation and differentiation during EG (45) Promotion of leukemogenesis by LIP isoform (44)

IRF8	Expression of proinflammatory cytokines (54) Macrophage differentiation (57) Termination of EG response (17) Tumor suppression (59)

STAT3	Important during G-CSF signaling (68) Constitutively active STAT3 in AML cell lines (71)

STAT5	Anti-apoptotic role during myeloid differentiation (98) Oncogenesis (99)

*AML, acute myeloid leukemia; EG, emergency granulopoiesis; G-CSF, granulocyte colony stimulating factor; HSC, hematopoietic stem cell*.

## HOXA10-Role in Immune Cell Development and Leukemia

HOXA10 is a homeodomain-containing TF which is a part of the A cluster on chromosome 7. HOXA10 is considered a master regulator of postnatal hematopoietic development that controls hematopoietic stem cell (HSC) self-renewal, the development of lymphoid and erythroid/megakaryocyte cells, as well as platelet biogenesis (19–21). It is also abundantly expressed in myeloid progenitors, where it influences myelopoiesis (22–24) and in phagocytic cells, where it represses transcription of the genes encoding p67^phox^ (NADPH oxidase subunit) and gp91^phox^ (cytochrome b subunit beta), thereby influencing its effector functions (25). However, overexpression of HOXA10 in murine bone marrow has been shown to induce a myeloproliferative disorder (MPD) involving expansion of the committed myeloid progenitors, which later evolves into acute myeloid leukemia (AML) (26). Leukemias with chromosomal translocations of the mixed lineage leukemia 1 (*MLL1*) gene are characterized by increased and sustained transcription of a group of *HOX* genes (including *HOXA10*), as fusion proteins generated by *MLL1* gene translocations lack ubiquitination/degradation domains (27). Mice transplanted with bone marrow expressing an MLL-fusion protein or overexpressing HOXA10 develop AML (26, 28–30). In a recent study, the MLL-ELL fusion protein was found to increase expression of HOXA9 and HOXA10 directly, by interaction with their promoters, and indirectly via fibroblast growth factor 2 (FGF2), β-catenin, and caudal-type homeobox 4 (CDX4) (31).

## HOXA10-Role in Emergency Granulopoiesis through HOXA10-TRIAD1 Interaction

Apart from the leukemogenic role of HOXA10, its role in regulating EG has also been elucidated recently. HOXA10^−/−^ mice showed a fatal EG response, which was rescued by re-expression of TRIAD1 (alias ARIH2) (32). TRIAD1, encoded by the gene *ARIH2*, is a ubiquitin ligase that regulates myelopoiesis by inhibiting proliferation of myeloid cells (32, 33). In one study, hematopoietic deficiency of ARIH2 caused lethal activation of the immune system. Sustained activity of NF-κB TF subunit p65 (RELA) was found in the nucleus of *ARIH2*-deficient dendritic cells, which caused lethal immunological responses in *ARIH2*-sufficent mice reconstituted with *ARIH2*-deficient hematopoietic stem cells (34). *ARIH2* has been shown to be a target gene for HOXA10, with the tandem *cis* elements in the *ARIH2* promoter being activated by HOXA10. *In vitro* stimulation of myeloid progenitor cells with G-CSF showed HOXA10-dependent increase in TRIAD1 expression (33). As G-CSF is the prime mediator of EG, this study implicates EG-driven up-regulation of TRIAD1 by HOXA10 and presents protein ubiquitination/degradation as a novel mechanism of regulating EG response by HOXA10. Increased TRIAD1 expression degrades FGFR1, thereby reducing the levels of FGF2 and terminating the effect of FGF2 on myeloid progenitor expansion and phagocyte effector function. All these processes culminate in termination of EG, with HOXA10 being the prime mediator. Moreover, in the bone marrow of HOXA10^−/−^ mice, TRIAD1 expression was only slightly decreased at steady state but TRIAD1 expression was totally absent during EG, suggesting the specific role of HOXA10 during EG. Transcription of *ARIH2* and expression of TRIAD1 during EG was regulated by tyrosine phosphorylation of HOXA10. Thus, this study elucidated the induction of protein degradation via TRIAD1 as a novel immune modulatory mechanism of HOXA10 (32).

**Figure 1:**
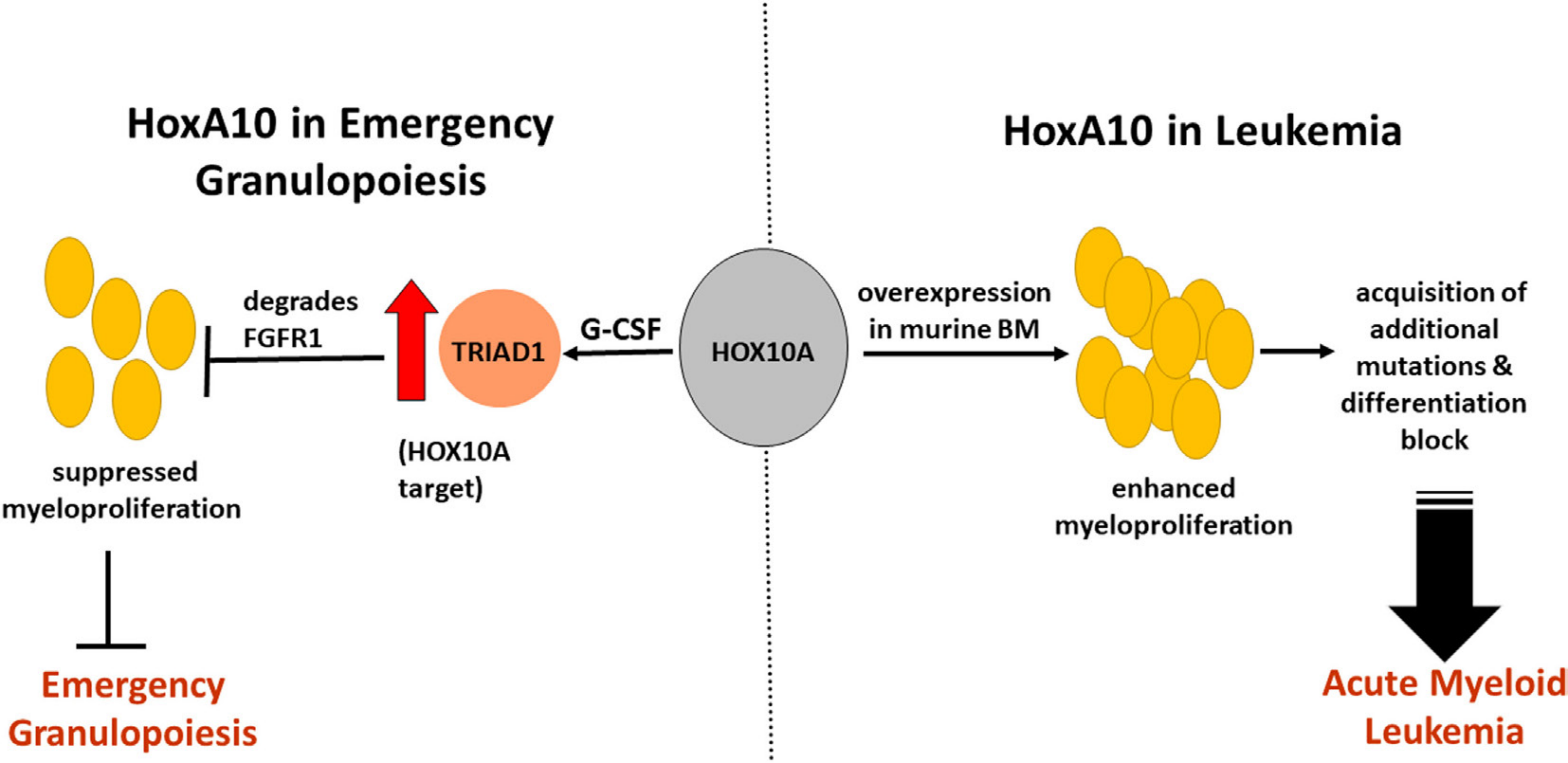
Impact of HOXA10 on acute myeloid leukemia (AML) development and emergency granulopoiesis (EG) through regulation of myelopoiesis. HOXA10 overexpression in murine bone marrow leads to enhanced myeloproliferation, which progresses into AML through acquisition of additional mutations and differentiation block. HOXA10 effects EG by regulating the gene *ARIH2*, which encodes TRIAD1, a ubiquitin ligase. *In vitro* stimulation of myeloid progenitors with granulocyte colony stimulating factor (G-CSF) showed HOXA10-dependent increase in TRIAD1 expression. Increased TRIAD1 expression degrades fibroblast growth factor receptor-1 (FGFR1), reduces the effect of FGF2, and terminates the effect of FGF2 on myeloid progenitor expansion. As G-CSF is the main mediator of EG, these processes result in termination of EG with HOXA10 being the prime regulator. Suppressed EG response during infections in leukemic conditions may serve as one of the causes of neutropenia.

Based on these studies, it can be speculated that, during EG in leukemia, overexpression of HOXA10 leads to sustained activation of TRIAD1, which favors a suppressed EG state, thus identifying one factor that may cause neutropenia in leukemia patients and make them more susceptible to infections (Figure 1). On the other hand, higher leukemia transformations have been reported in severe congenital neutropenia (SCN) patients who required higher G-CSF doses (35), indicating that there are common TFs that mediate leukemogenesis and granulopoiesis.

## CCAAT/Enhancer Binding Protein Beta—Role in Immune Cell Development and Leukemia

CCAAT/Enhancer Binding Protein beta (C/EBP-β) is a basic leucine zipper (bZIP) domain-containing TF that plays an important role in regulating immune and inflammatory responses (36–39). Both leukemia suppressor and pro-oncogenic roles of C/EBP-β have been reported. C/EBP-β was shown to suppress the leukemogenic potential of 32D-BCR-ABL cells by inducing granulocytic differentiation and by inhibiting cell proliferation. Low C/EBP-β expression is observed in the blast crisis stage of chronic myelogenous leukemia (CML) and is inversely correlated with BCR-ABL tyrosine kinase levels, suggesting that there may be therapeutic potential in restoring its activity in CML-BC (40). In acute promyelocytic leukemia (APL), treatment with all-trans retinoic acid (ATRA) reverses promyelocytic leukemia-retinoic acid receptor α (PML-RARα)-mediated differentiation block at the promyelocyte stage resulting in mature neutrophil-like cells (41, 42). C/EBP-β is upregulated in the presence of PML-RARα during ATRA treatment and promotes the proliferation and differentiation of APL cells, thereby showing a potential anti-cancer role (42).

C/EBP-β exists as several isoforms due to alternative translation initiation: full-length C/EBP-β liver activating protein* (LAP*), a slightly shorter isoform of LAP that lacks the first 21 amino acids and a short isoform of liver inhibitory protein (LIP). LAP* and LAP are trans-activators, whereas LIP is a transcriptional repressor. The relative abundance of LIP and LAP C/EBP-β isoforms mediated through the regulation of translation initiation is important in determining cell fate by controlling proliferation and differentiation (43). In contrast to the leukemia suppressive effect of C/EBP-β, its LIP isoform was shown to promote leukemogenesis in a mouse bone marrow transplantation system by collaborating with Ecotropic viral integration site 1 (*Evi1*) which is one of the master regulators of AML development. However, experiments performed on human whole BM cells from AML patients revealed that *Evi1* closely correlated with both LAP* and *LIP* expression (44).

## CCAAT/Enhancer Binding Protein Beta—Role in Emergency Granulopoiesis

Hirai et al. showed that C/EBP-α is required for steady-state granulopoiesis whereas C/EBP-β is essential for EG (45–47). Only C/EBP-β expression (and not that of other C/EBPs) was upregulated in GMPs after cytokine treatment (45). Using *C/EBP* β^−/−^ bone marrow cells, it was found that C/EBP-β is involved in cytokine (G-CSF, GM-CSF, IL-3, and IL-6)-induced myeloid proliferation, suggesting that C/EBP-β is required to couple proliferation and differentiation of granulocytes under stress or emergency situations, thereby producing more mature granulocytes (45). The specific role of LAP*, LAP, and LIP isoforms of C/EBP-β in EG is not clear; however, exploring this may help target the specific isoform for both anti-leukemic and anti-neutropenic effects.

## IRF8 (Also known as Interferon Consensus Sequence Binding Protein)—Role in Immune Cell Development and Leukemia

The critical role of IRF8 in innate immune response and oncogenesis has been described extensively (48–51). In response to pattern-recognition receptors (PRR) activation, IRF8 induces the expression of proinflammatory cytokines through TLR9-MyD88-dependent signaling (52–55). IRF8 inhibits cell growth and promotes apoptosis in myeloid cells and drives their differentiation toward macrophages while inhibiting neutrophil production (56, 57). IRF8 plays an important role in myeloid cell development, as has been demonstrated by a systemic expansion of neutrophils followed by a fatal blast crisis, resembling human CML in *IRF8*^−/−^ mice (50). These mice also exhibit increased progenitor cell numbers with enhanced responsiveness to granulocyte-macrophage colony stimulating factor (GM-CSF) and G-CSF. In contrast, responsiveness to macrophage colony stimulating factor (M-CSF) was reduced in *IRF8*^−/−^ progenitors, implying a role for IRF8 in driving toward the macrophage lineage (57, 58). A tumor suppressor role has been described for IRF8, as very low or absent *IRF8* mRNA was found in the peripheral blood of the majority of human myeloid leukemias. Sorted B-cells derived from CML patients also showed the absence of *IRF8* mRNA (59). Moreover, high *IRF8* mRNA levels were found only in those CML patients who were classified as “good” cytogenetic responders to interferon-α therapy and not in the poor responders (60). One of the target genes of IRF8 is Fas-associated phosphatase 1 (*FAP1*; the *PTPN13* gene), which shares a reciprocal expression profile with *IRF8* at all clinical stages of CML. Impaired *IRF8* expression in *BCR−ABL* + myeloid progenitor cells contributed to FAP1-dependent Fas resistance. As Fas resistance contributes to persistence and expansion of CML leukemic stem cells (LSCs), it led to imatinib resistance in *BCR − ABL* + GMPs through the reduction of IRF8 expression and increased FAP1 expression (61).

IRF8 also represses the Growth Arrest Specific 2 gene (*GAS2*), which encodes a calpain inhibitor that is involved in cell proliferation and survival. *GAS2* expression in *BCR-ABL*^+^ cells stabilizes β-catenin, which is a calpain substrate (62). In addition to β-catenin, calpain has other substrates that are involved in the pathogenesis of CML including signal transducer and activator of transcription 5 (STAT5) (63). In a recent study, BCR-ABL-induced SHP2-dependent dephosphorylation of IRF8 was found to impair repression of *GAS2*, leading to decreased calpain activity and thereby an increase in its substrate protein STAT5, which in turn represses *IRF8* promoter. This novel feedback mechanism involving calpain enhances leukemogenesis by increasing STAT5 and repressing IRF8. Hence, therapeutic upregulation of IRF8 can reduce persistent LSCs during treatment of CML with BCRABL-targeted tyrosine kinase inhibitors (TKIs) (64). In another study, induction of IRF8 and repression of β-catenin was found upon arachidonate 15-lipoxygenase (15-LO) inhibition by PD146176 in K562 cells, implicating another mechanism where IRF8 may be involved in eradicating CML LSCs (65). In addition to the role of IRF8 in myeloid leukemia, recently its role in suppressing acute lymphoblastic leukemia has been described. Mice deficient for both *PU.1* and *IRF8* developed pre-B-ALL at high frequency by reducing the expression of known tumor suppressors, including SPI-B, IKAROS, and BLNK (66).

## IRF8 as a Regulatory Component of Emergency Granulopoiesis

**Figure 2:**
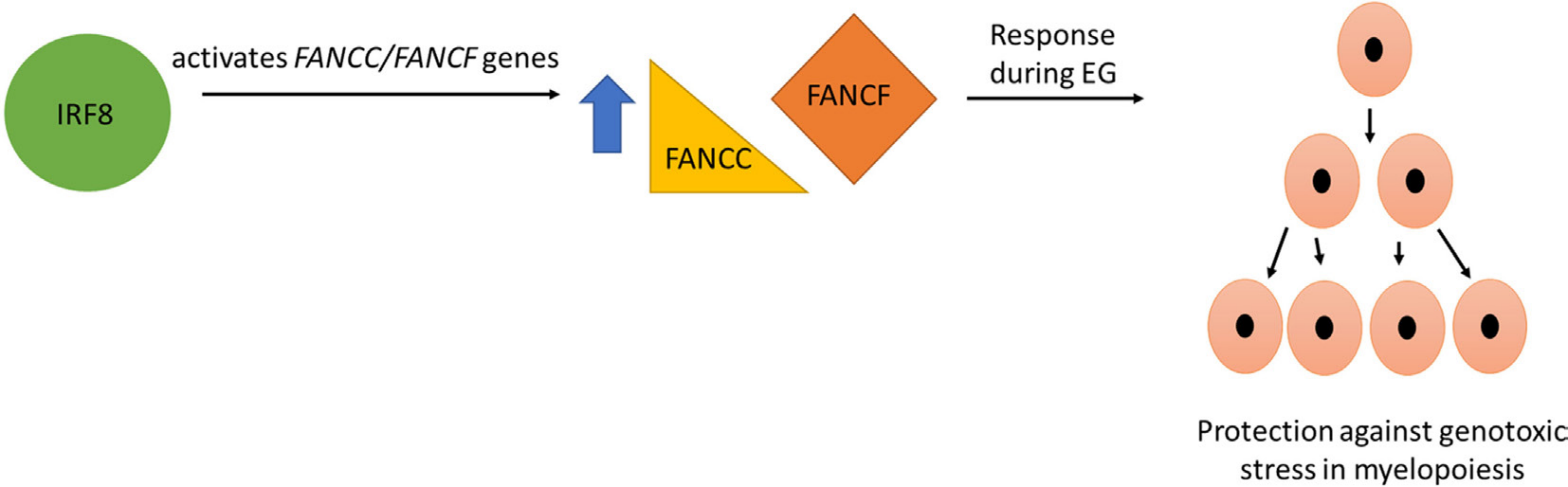
IRF8 plays an important role in myeloid cell development during emergency granulopoiesis (EG) via regulating Fanconi DNA repair proteins. Some of the target genes of IRF8 include *FANCC* and *FANCF*, encoding Fanconi C and Fanconi F, respectively. These Fanconi proteins contribute to Fanconi DNA repair pathway activation during EG and protect cells from the genotoxic stress of myelopoiesis during rapid proliferation phase.

In addition to its leukemia suppressor role, IRF8 plays an important regulatory role in innate immune response (51). Some of the target genes of IRF8 include *FANCC* and *FANCF*, encoding Fanconi C and F, respectively, which contribute to the Fanconi DNA repair pathway activation during infectious challenge (16, 67). IRF8 activates *FANCF cis* element in differentiating myeloid cells, thereby protecting them from genotoxic stress associated with differentiation (67). IRF8 was also found to bind and activate a *cis* element in the proximal *FANCC* promoter. Re-expression of FANCC rescued DNA repair in IRF8-deficient myeloid cells. Furthermore, IRF8 activates *FANCC* in murine myeloid progenitor cells upon stimulation with IL-1β and G-CSF, cytokines that are essential for EG (16). As rapid expansion due to S-phase shortening of granulocyte/monocyte progenitor (GMP) populations occurs during the cell cycle, IRF8 contributes to genomic stability during EG through the Fanconi pathway (16, 67) (Figure 2).

**Figure 3:**
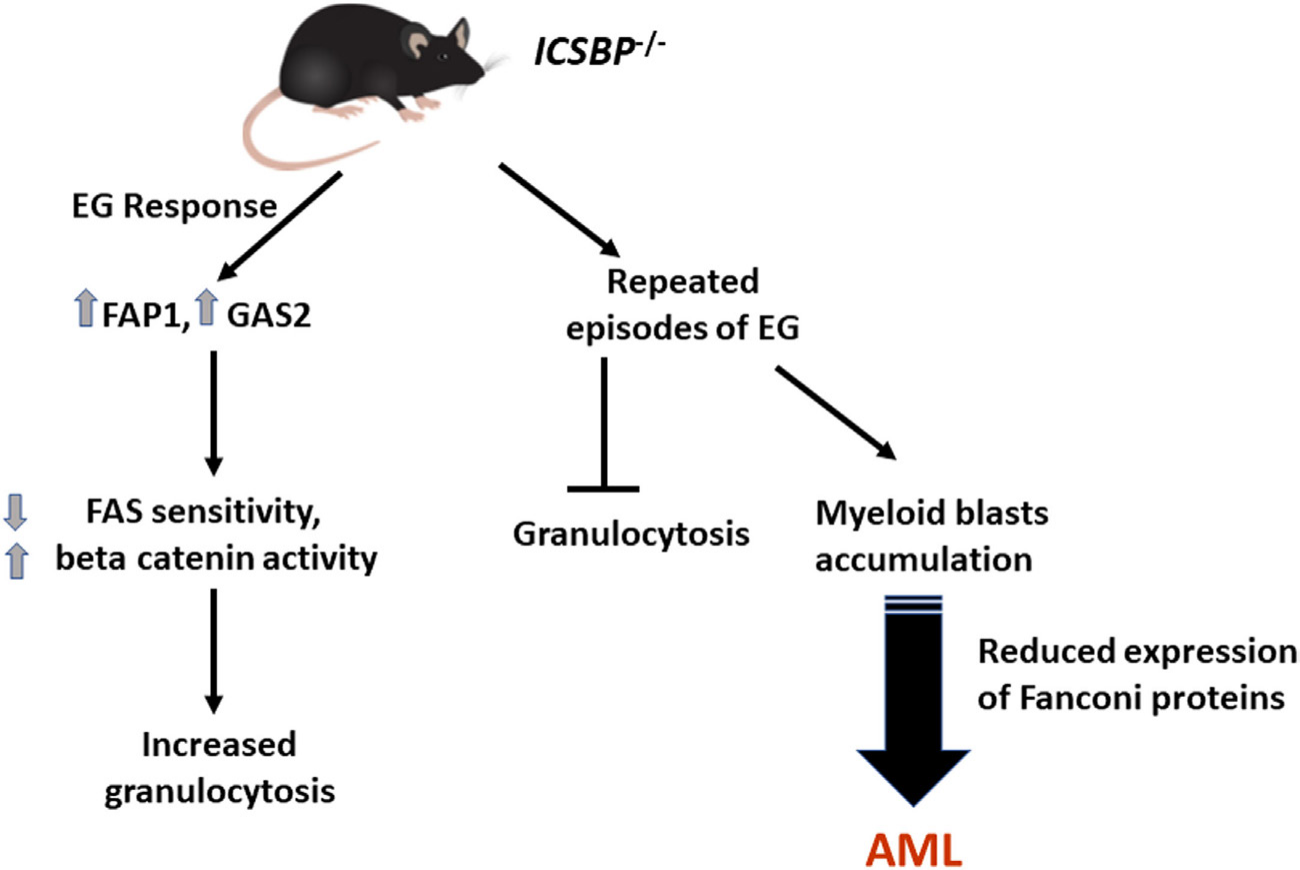
Repeated episodes of emergency granulopoiesis (EG) lead to leukemia development in *IRF8*^−/−^ (*ICSBP*^−/−^) mice. A sustained expansion of neutrophils was observed in *IRF8*^−/−^ mice in response to EG through increased expression of FAP1 and GAS2 in bone marrow myeloid progenitors. However, repeated episodes of EG did not increase granulocytes in *IRF8*^−/−^ mice, but accumulation of myeloid blasts was found, leading to acute myeloid leukemia (AML) development. Reduced expression of FANCC and FANCF, which are IRF8 target proteins, contribute by increasing sensitivity to DNA damage in bone marrow myeloid progenitors.

In addition to this, a more specific role of IRF8 in the termination of EG response was recently described. In *IRF8^−/−^* mice, sustained granulocyte production was found in response to EG via increased expression of FAP1 and GAS2 in bone marrow myeloid progenitor cells, which leads to decreased FAS sensitivity and increased β-catenin activity in these cells. This implies that IRF8 mediates termination of the EG response by increasing FAS-induced apoptosis and decreasing β-catenin activity in these cells, thereby limiting granulocyte proliferation (17). However, repeated episodes of EG did not increase granulocytes in *IRF8*^−/−^ mice; instead, an accumulation of myeloid blasts was found leading to AML development (Figure 3). This effect is mediated through reduced expression of IRF8 target proteins FANCC and FANCF with increased sensitivity to DNA damage in bone marrow myeloid progenitors (17).

From these observations, it can be inferred that there may be a dual impact of reduced IRF8 on neutrophil production in leukemic conditions during an EG response. Reduced IRF8 would prolong the termination step of EG (by increasing FAP1 and GAS2 expression), thereby causing granulocytosis with functionally incompetent cells (17). Simultaneously, it will also promote apoptosis of myeloid progenitors during the rapid proliferation step via decreased expression of FANCC and F that rescue collapsed and stalled replication forks during DNA replication. This leads to reduced neutrophil count or enhanced leukemogenesis due to accumulation of mutations. Therefore, gene regulation of Fanconi DNA repair proteins by IRF8 presents an important link between innate immune response and the leukemia suppressor role of IRF8.

## STAT Proteins

### STAT3—Role in Immune Cell Development and Leukemia

STAT3 is one of the most important TFs phosphorylated by Janus kinases after G-CSF-induced dimerization of G-CSFR (68, 69). G-CSF and IL-6 are potent activators of STAT3 in hematopoietic progenitor cells and neutrophils (69, 70). Constitutively active STAT3 has been reported in human AML cell lines and in pretreatment bone marrow samples of AML patients, where it was associated with shorter disease-free survival (71, 72). Inhibiting the G-CSF-induced STAT3 phosphorylation by a small-molecule STAT3 inhibitor C188-9 induced apoptosis in AML cell lines and primary pediatric AML samples (73). Another STAT3 inhibitor, BP-5-087, in combination with imatinib, reduced the survival of primary tyrosine kinase inhibitor (TKI)-resistant stem and progenitor cells, as activation of pSTAT3 (at amino acid position Y705) is important for BCR-ABL1 kinase-independent TKI resistance (74).

STAT3 interacts directly with c-Jun, as has been reported using the yeast two-hybrid system (75). STAT3, in cooperation with c-JUN and c-FOS, activates the IL-6 response element (IRE) (76). Elevated c-JUN expression has been linked to AML, where it inactivates *C/EBP-α* via leucine zipper domain interaction. This interaction and C/EBP-α inactivation are necessary to induce proliferation in AML (77). The role of c-JUN during EG has not been explored yet; however, its interaction with STAT3 and IRE activation point toward its involvement as a positive regulator of EG. c-JUN may act as an additional TF that lies at the intersection of EG and leukemia and may enhance the development of leukemia along with skewing of the EG response.

### STAT3—Role in Emergency Granulopoiesis

In response to G-CSF, STAT3 accelerates granulocyte cell-cycle progression (G1–S phase transition) and terminal maturation by regulating C/EBP-β, which is an important TF in the EG response. STAT3 and C/EBP-β co-regulate *c-MYC* through direct interaction with its promoter and displacement of C/EBP-α during demand-driven granulopoiesis (78). A recent study has shown the involvement of STAT3 and C/EBP-β in the activation of *FANCC* transcription which contributes to DNA repair during EG (79).

## STAT3 Regulates Suppressor of Cytokine Signaling-3 (SOCS3) Expression

Studies done in STAT3-deficient mice show neutrophilia and hyper-responsiveness of myeloid cells to G-CSF. This was related to reduced SOCS3 expression, thereby showing the role of STAT3 in inducing SOCS3, which in turn acts as a negative regulator of proliferative signals from G-CSF signaling required during EG (80). SOCS3 deficiency significantly increases STAT3 activation in response to *in vivo* administration of G-CSF which leads to toxic effects (81). Similarly, in another study on *SOCS3*-deficient hematopoietic progenitor cells, STAT3 and C/EBP-β activation was increased in response to G-CSF and IL-6 (82). As mentioned above, STAT3 induces C/EBP family TFs and interacts with them to augment the effect of G-CSF signaling (83). SOCS3 is recognized as a negative regulator of G-CSF signaling and EG in myeloid cells (81, 84). Constitutive STAT3 phosphorylation was shown to constitutively activate SOCS3 expression in AML blasts isolated from patients (85). An inefficient EG response during infectious episodes can be expected from constitutively increased expression of SOCS3 under such leukemic conditions. Another study on STAT3-deficient mice showed a failure to accumulate immature granulocytes in the bone marrow after G-CSF exposure, thereby leading to an increase in the ratio of mature to immature neutrophils. However, immature granulocytes are needed during EG for increased neutrophil production. The study characterized impairment in acute neutrophil mobilization which is independent of SOCS3, indicating diverse signaling pathways in response to G-CSF (86).

Recently, activating somatic STAT3 mutations located in the Src homology 2 (SH2) domain have been described in T-cell large granular lymphocytic leukemia with a high frequency of 40% (31 of 77 patients). These patients presented more often with neutropenia than patients without these mutations (87). As discussed earlier, this could be due to the induction of SOCS3 by activated STAT3, which negatively regulates G-CSF signaling (81). Based on these observations, constitutive activation of STAT3 might contribute to failure of the EG response in leukemic patients resulting in neutropenia and increased susceptibility to infections.

Targeting STAT3 can provide highly specific approach to treat failed EG responses in leukemia. Evidently, there is a growing list of STAT3 inhibitors under clinical evaluation which rely on direct or indirect targeting mechanisms (88). Most common STAT3 targeting approaches include inhibition of tyrosine kinases that phosphorylate and upregulate STAT3 activity (89). Others include STAT3 SH2 domain (dimerization) inhibitors thereby preventing the formation of functional STAT3 dimers; STAT3 DNA binding domain inhibitors that prevent binding of STAT3 to its DNA promoter, *STAT3* gene expression oligonucleotide inhibitors which compete for STAT DNA binding (88), and STAT3 N-terminal domain inhibitors that disrupt protein–protein interactions between STAT3 and other TFs (90). Therefore, in addition to the possible therapeutic potential of STAT3 inhibitors in cancer treatment they can also be considered for their efficacy in treating infectious episodes in leukemia.

## STAT5—Role in Immune Cell Development and Leukemia

STAT5 is an important STAT family protein that is activated by G-CSF (91). STAT5a and STAT5b are two forms of STAT5 that are encoded by two distinct but closely related genes (92) and are activated by tyrosine phosphorylation through many factors and cytokines like prolactin, growth hormone (93), erythropoietin (94), thrombopoietin (95), interleukin 3 (IL-3), GM-CSF (96), and interleukin 2 (IL-2) (97). An anti-apoptotic role of STAT5 has been documented during differentiation of myeloid progenitors. In a study by Kieslinger et al. (98), primary chicken myeloblasts expressing dominant-negative alleles of STAT5 were unable to generate mature cells due to increased apoptosis during differentiation. Bone marrow cells from STAT5a/STAT5b-deficient mice showed increased apoptosis during GM-CSF-dependent maturation *in vitro*. This apoptotic cell death was rescued by ectopic expression of BCL-X, thereby showing an important role of STAT5 during cytokine-dependent differentiation of myeloid progenitors during inflammation (98).

STAT5 signaling can promote oncogenesis (99) and hyperactivated STAT5 has been implicated in various leukemia types such as BCR-ABL-induced CML and AML, and in MPDs, such as chronic myelomonocytic leukemia and polycythemia vera (99–101).

Constitutive STAT5 activation has been demonstrated to be essential in a mouse model of MPD induced by TEL-JAK2 fusion protein. TEL-JAK2 fusion protein-mediated constitutive STAT5 activation is essential in a mouse model of MPD (102). Constitutively active STAT5 mutant in CD34^–^c-Kit^+^Sca-1^+^ lineage marker^−^ (CD34^–^KSL) HSCs induced fatal MPD in a mouse model, implying the crucial role of STAT5 in self-renewal of HSCs during MPD development (99). These studies show that STAT5 is involved in both neutropenic conditions and development of hematologic malignancies. Therapeutic approaches to target caSTAT5 are being studied, like the small molecule bromodomain inhibitor JQ1, which reduces STAT5 function in leukemia and lymphoma cells with caSTAT5 (103).

## STAT5-Possible Regulatory Role in Emergency Granulopoiesis

STAT5-null (STAT5A and STAT5B) HSCs in mice show an impaired repopulation potential and disrupt multilineage hematolymphoid development in the bone marrow, including a reduction in neutrophil progenitors and mature neutrophils (104). However, constitutively active STAT5 (caSTAT5a) and not wild-type STAT5a is associated with inhibition of lymphoid enhancer-binding factor 1 (LEF-1) in CD34 + cells of congenital neutropenia (CN) patients. LEF-1 positively regulates G-CSF triggered granulopoiesis by promoting proliferation and differentiation of granulocyte precursors (105). caSTAT5a inhibits LEF-1-dependent autoregulation of *LEF-1* gene promoter by binding to the LEF-1 protein, recruiting Nemo-like kinase and the E3 ubiquitin-ligase NARF to LEF-1. This leads to LEF-1 ubiquitination and a reduction in LEF-1 protein levels, severely affecting neutrophil production. Interestingly, sustained elevation of phospho-STAT5 in CD34 + cells was observed in the CN patients compared to healthy controls, which was correlated to the development of AML (100).

## Emerging Role of Fanconi Anemia (FA) DNA Repair Pathway Interconnects Leukemia and Innate Immune Response

To date, 19 genes belonging to FA complementation groups are known (A, B, C, D1, D2, E, F, G, I, J, L, M, N, O, P, Q, R, S, T) (106, 107). The FA pathway is required to repair DNA interstrand crosslinks (ICLs) which involves nucleotide excision repair (NER), translesion synthesis (TLS), and homologous recombination (HR) (108). ICLs affect DNA replication and transcription through stalling of replication forks and preventing strand separation. Therefore, unrepaired ICLs lead to DNA breakage and chromosomal rearrangements resulting in cellular apoptosis or accumulation of mutations (109). In this regard, FA pathway plays an important role in genome maintenance by repairing DNA damage during replication stress responses, especially during S phase of the cell cycle (110). Hypersensitivity to DNA damage agents that induce ICL is observed in cells deficient in any component of FA pathway (111, 112). Cells undergo G2/M arrest and chromosomal breakage on treatment with mitomycin C (MMC) or diepoxybutane (DEB) (113, 114). In humans, germline inactivation of any FA gene predisposes them to increased sensitivity to ICLs, thereby resulting in bone marrow failure (BMF) and cancer development (115–117).

The FA pathway is also important to maintain hematopoietic stem and progenitor cells (HSPC) population. In FA patients, p53/p21 activation and G0/G1 cell cycle arrest occurs in HSPC leading to BMF in FA, whereas p53 deficiency rescued the HSPC defects in human FA cells. Therefore, HSPC instability in FA patients increases their chances to develop AML (118). K-RAS or c-MYC induced oncogenic stress caused a short-lived response in mice deficient for the FA core complex components FANCA or FANCC. Downregulation of Protein Arginine Methyltransferase 5 (PRMT5) led to compromised K-ras^G12D^-induced arginine methylation of p53 in *FANCA* deficient cells, thereby demonstrating an arginine methylation-dependent FA-p53 interaction, as forced expression of PRMT5 in *FANCA^−/−^* HSPCs prolonged oncogenic response and delayed leukemia development in irradiated recipient mice (119). In another recent study, *FANCC* deficient aging mice developed hematologic malignancies that precede genomic instability and hematopoietic chromosomal instability or aneuploidy (120). This is further supported by the observation that AML displays an acquired decrease in expression of Fanconi proteins. Role of FANCF in leukemia suppression has also been shown. CHRF-288 (an AML cell line) exhibits a cellular FA phenotype due to lack of FANCF expression, which is corrected by a *FANCF*-expressing plasmid. *FANCF* is localized in a hot-spot region for somatic hypermethylation (11p15); therefore, gene silencing due to hypermethylation of the promoter region of the *FANCF* gene explains the absence of FANCF protein in CHRF-288 cell line (121). *FANCF* is also an *IRF8* target gene that provides genomic stability to myeloid cells from DNA cross-link damage during proliferation and differentiation stages (67). Mitomycin C-induced DNA damage was increased in *IRF8* deficient primary murine bone marrow cells, which was rescued by FANCF overexpression (17). Together, these findings strongly suggest a functional cross-talk between cell proliferation and DNA repair pathways.

Until now, the major roles of Fanconi pathway have been shown in maintenance and proliferation of HSPC (122); tumor suppression (123–127); stabilizing the replication fork during S phase; and DNA repair processes to protect against unwanted mutations (108, 128–130). However, its role in innate immune response has emerged recently. As mentioned previously, *FANCC* is an IRF target gene and *IRF8*^−/−^ mice succumbed to infectious challenge with failed leukocytosis response (16, 50, 131). This observation implies that EG will be impaired in response to infectious or inflammatory challenge. As Fanconi proteins protect cells from genotoxic stress of myelopoiesis during rapid proliferation phase, their potential role in EG response is plausible. *FANCC*-deficient mice showed an abnormal response to EG and developed progressive neutropenia and anemia which resulted from excess apoptosis of bone marrow HSCs and myeloid progenitors. These effects led to failed EG episodes which contributed to BMF and suggest that FANCC expression and Fanconi pathway is enhanced during infectious episodes and is an essential element of EG response. Upon treatment with an essential EG cytokine IL-1β, *FANCC, FANCJ*, and *RAD51* were enriched in the chromatin fraction of murine myeloid progenitor cells, signifying their increased expression and diverse DNA repair processes initiated during EG. Moreover, treatment with an IL-1R antagonist (anakinra) in alum-treated FANCC-knockout mice ameliorated BMF (16).

These observations again point toward the overlapping role of FANCC protein in leukemia suppression and completing a successful EG response. Lack of this protein resulted in enhanced susceptibility to AML development and failed EG in response to infectious challenge thereby leading to neutropenia (16).

## Conclusion

From the studies reviewed here, it can be inferred that the alterations in the expression of TFs which promote leukemia also cause an improper EG execution leading to neutropenia. Neutropenic conditions will result in increased susceptibility to infections. That is why severe sepsis is observed in cancer patients at a much higher rate than in non-cancer patients (132). So, a counter question can also arise: do neutropenic conditions lead to leukemia? An example can be found in myelodysplastic syndromes where the neutropenic patients are at a higher risk for leukemia. The findings, albeit very few, point to a role of TFs in the pathogenesis of these diseases and it will be of interest to study if failed EG episodes predisposes a person toward leukemia. Identifying TFs that can revert the disease phenotype or selectively treat neutropenia in conjunction with drugs used for leukemia is another highly promising strategy that may be tested. Therefore, targeting intersecting TFs can be of therapeutic value to treat lymphoid and myeloid leukemia and associated disorders including neutropenia.

## Author Contributions

SH designed and drafted the manuscript. SH and ARN wrote the manuscript. AR and SH finalized the figures and table. All authors read and approved the final manuscript.

## Conflict of Interest Statement

The authors declare that there is no conflict of interest, be it financial, commercial, or other.

## Abbreviations

EG, emergency granulopoiesis; TFs, transcription factors; HSC, hematopoietic stem cells; AML, acute myeloid leukemia; MLL1, mixed lineage leukemia 1; FGF2, fibroblast growth factor 2; IRF8, interferon regulatory factor; C/EBP-β, CCAAT/enhancer binding protein beta; ICSBP, interferon consensus sequence binding protein; GAS2, growth arrest specific 2; LSCs, leukemic stem cells; FAP1, Fas-associated phosphatase 1; FANCC, Fanconi C; FANCF, Fanconi F; SOCS3, suppressor of cytokine signaling-3; caSTAT5, constitutively active STAT5; MPD, myeloproliferative disorder; BMF, bone marrow failure; FA, Fanconi anemia.
